# Test–Retest Reliability of Measures Commonly Used to Measure Striatal Dysfunction across Multiple Testing Sessions: A Longitudinal Study

**DOI:** 10.3389/fpsyg.2017.02363

**Published:** 2018-01-12

**Authors:** Clare E. Palmer, Douglas Langbehn, Sarah J. Tabrizi, Marina Papoutsi

**Affiliations:** ^1^Sobell Department of Motor Neuroscience and Movement Disorders, Institute of Neurology, University College London, London, United Kingdom; ^2^Carver College of Medicine, University of Iowa, Iowa City, IA, United States; ^3^Huntington’s Disease Centre, Institute of Neurology, University College London, London, United Kingdom

**Keywords:** reliability, longitudinal, cognitive function, cognitive impairment, inhibitory control, emotion recognition, striatal impairment

## Abstract

Cognitive impairment is common amongst many neurodegenerative movement disorders such as Huntington’s disease (HD) and Parkinson’s disease (PD) across multiple domains. There are many tasks available to assess different aspects of this dysfunction, however, it is imperative that these show high test–retest reliability if they are to be used to track disease progression or response to treatment in patient populations. Moreover, in order to ensure effects of practice across testing sessions are not misconstrued as clinical improvement in clinical trials, tasks which are particularly vulnerable to practice effects need to be highlighted. In this study we evaluated test–retest reliability in mean performance across three testing sessions of four tasks that are commonly used to measure cognitive dysfunction associated with striatal impairment: a combined Simon Stop-Signal Task; a modified emotion recognition task; a circle tracing task; and the trail making task. Practice effects were seen between sessions 1 and 2 across all tasks for the majority of dependent variables, particularly reaction time variables; some, but not all, diminished in the third session. Good test–retest reliability across all sessions was seen for the emotion recognition, circle tracing, and trail making test. The Simon interference effect and stop-signal reaction time (SSRT) from the combined-Simon-Stop-Signal task showed moderate test–retest reliability, however, the combined SSRT interference effect showed poor test–retest reliability. Our results emphasize the need to use control groups when tracking clinical progression or use pre-baseline training on tasks susceptible to practice effects.

## Introduction

Cognitive impairment is common in many neurodegenerative diseases that are often associated with motor impairment such as Parkinson’s disease (PD) and Huntington’s disease (HD; O’Callaghan et al., 2014). When evaluating sensitive biomarkers for disease progression two main factors need to be taken into consideration: sensitivity to disease progression and test–retest reliability. Sensitivity to disease progression is most commonly measured in longitudinal, observational trials of a clinical population with consecutive measurements at least a few months apart, depending on the progression rate of the disease of interest. Test–retest reliability is most-commonly evaluated in healthy controls with short or long intervals between observations. The main aim of the test–retest reliability measurements is to identify the stability of a given measure across repetitions. A measure that varies widely within-participants from 1 day to the next is not stable enough to be used as a biomarker. Therefore, before any task can be used to evaluate therapeutic interventions in any population, it is important to evaluate this. Moreover, for many cognitive tasks, participants improve over multiple testing sessions due to practice. Therefore, it is additionally imperative to determine whether a given task is vulnerable to practice effects. Tasks susceptible to practice may still be used to track disease progression, however, necessary steps must be taken to diminish any effects of practice and ensure they cannot confound any positive improvement recorded through the use of carefully selected control groups.

We focused on tasks that are commonly used as biomarkers in neurodegenerative diseases to measure cognitive dysfunction associated with striatal impairment. In more detail, we created modified versions of the Face Emotion Recognition task (based on the task used as part of the Track-HD battery; Ekman and Friesen, 1976; Labuschagne et al., 2013) and the Trail Making task (Reitan, 1958). We also tested a newly derived Circle Tracing measure: speed of tracing (Lemay et al., 2005). These tasks have been previously validated as biomarkers sensitive to disease progression in neurodegenerative diseases (e.g., PD: Uc et al., 2006; Inzelberg et al., 2008; Ricciardi et al., 2017; HD: Stout et al., 2011, 2012; and dementia: Diehl-Schmid et al., 2007; Ashendorf et al., 2008) and the modifications aimed to improve their suitability for longitudinal trials with patient populations. Lastly, we evaluated the suitability of a combined Simon Interference – Stop-Signal task. Although the Stop-Signal and Simon Interference tasks are commonly used separately as measures of inhibitory control, a novel study by Jahfari et al. (2011) showed that measures from the combined task are sensitive to striatal function, which is relevant for HD and PD pathology. The reliability of these tasks, however, has not been previously tested.

In this study, we aimed to evaluate the suitability of these selected tasks for longitudinal, interventional trials by recording participant performance across multiple testing sessions. To evaluate suitability for each task, we measured test–retest reliability in two ways: (1) by examining the presence of session effects, i.e., did the mean performance measure significantly change between testing sessions within-subjects; (2) by determining if the mean performance measure across subjects was significantly correlated between testing sessions. We also measured version effects (for tasks with multiple versions). For a measure to be considered suitable, we suggest it should meet the following criteria: (1) there should be no significant session differences at least between the second and third sessions; (2) there should be no significant version differences, otherwise the use of different versions would not be recommended; (3) the within-subject correlation between different sessions should be high.

Here we measured performance of 16 healthy subjects across three testing sessions on the four tasks mentioned and examined mean stability of task measures across testing sessions and how well those measures were correlated within-subjects to determine their test–retest reliability.

Although test–retest reliability of a task will be different between healthy participants and patients, if a task shows poor reliability in the control group it is unlikely that it will be better in the patient group, if anything it would probably be worse, given that patient groups tend to have larger inter-subject variability. The data from the control group can therefore help select the tasks and improve the design of longitudinal studies with a patient population.

## Materials and Methods

### Participants

Sixteen participants took part in the study (male = 8; female = 8). Each participant completed the combined Simon Interference – Stop-Signal task, the circle tracing task, the trail making test and the emotion recognition task once per session for three sessions. Mean test–retest interval between sessions 1 and 2 was 6.19 days (*SD* = 1.38, range 5–10 days) and between sessions 2 and 3 was 7.25 days (*SD* = 1, range 6–9 days). Participants ranged in age from 18 to 34 years with a mean age of 24 years (*SD* = 4.26). All participants were right-handed, had normal or corrected to normal vision and did not have any form of neurological disorder. All participants provided written informed consent in accordance with the Declaration of Helsinki. This study was carried out in accordance with the recommendations of the Queen Square Research Ethics Committee and the protocol was approved by the Queen Square Research Ethics Committee.

### Task and Procedure

#### Facial Emotion Recognition Task

**FIGURE 1:**
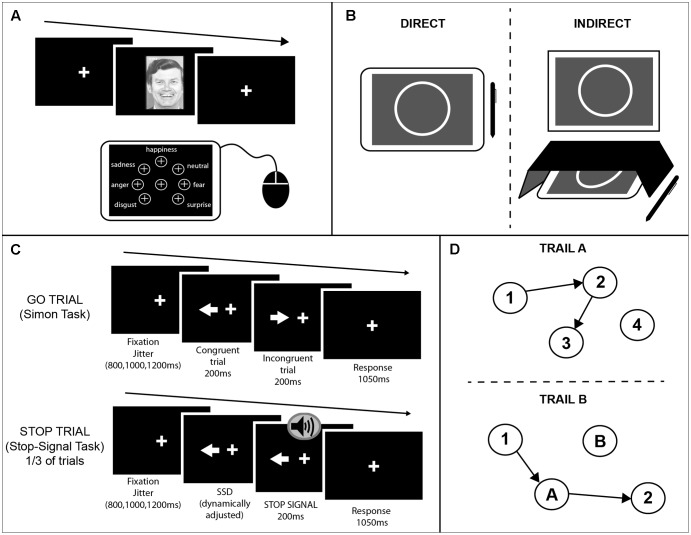
Schematics of the cognitive tasks used. **(A)** *Emotion recognition task.* A face was presented on an external monitor positioned in front of the participants. On each trial subjects were instructed to identify the emotion of the face presented on the screen and select one of seven options that appeared on the tablet screen using a mouse. The buttons for each of the seven emotions were arranged in equal distance away from the center button. **(B)** *Circle tracing task.* Participants were instructed to use a stylus to trace a white circle on a gray background. For the direct condition **(Left)** participants were instructed to trace the circle on the tablet screen. For the indirect condition **(Right)** a box was placed over the tablet so the participants could not see their hand; the circle was projected onto an upright monitor in front of the participant. **(C)** *Combined Simon-Stop-Signal Task.* A white arrow was presented on an upright monitor either side of the fixation cross pointing in the left or right direction. Participants were instructed to respond with their left or right index finger to the direction in which the arrow was pointing and ignore the location of the arrow (Simon task). On congruent (C) trials the arrow pointed in the same direction as the location in which it appeared on the screen. On incongruent (IC) trials the arrow pointed in the opposite direction to the side of the screen it appeared. On 30% of trials, an auditory stop-signal was given and subjects were instructed to inhibit their response [stop trials; Stop-Signal Task (SST)]. **(D)** *Trail Making Test.* A modified computerized version of the PEBL was administered using a touchscreen and stylus. The test included two parts both of which consisted of 25 circles randomly scattered over the touchscreen surface. In part **A (Upper)** these circles were numbered 1–25 and participants were instructed to tap inside the circles in ascending order using the stylus. In part **B (Lower)** the circles included numbers (1–13) and letters (A–L). Participants were instructed to tap inside the circles in ascending order alternating between numbers and letters (1–A–2–B, etc.). Three different versions of each part were used and counterbalanced across participants and sessions.

For this task, participants viewed pictures of faces expressing different emotions. The faces presented were from the Ekman 60 Faces test including the neutral condition (details of the stimuli used are mentioned in Labuschagne et al., 2013). The pictures were presented on an external monitor positioned in front of the participants. On each trial subjects were instructed to identify the emotion of the face presented on the screen and select one of seven options that appeared on the tablet screen using a mouse (see **Figure 1A** for the setup). The seven possible emotions were: neutral, happiness, anger, sadness, disgust, fear, and surprise. At the start of each trial the mouse cursor would move to the center of the tablet automatically. The buttons for each of the seven emotions were arranged in equal distance away from the center button. To make a response the participant had to move the cursor to the button corresponding to the chosen emotion and left-click. Using this setup ensured that there would be no positional bias when analyzing reaction times. A pseudo-randomized vector was created that determine the order of stimulus presentation. This was the same between sessions. To minimize practice effects, the vector only determined the type of emotion to be presented, whereas the specific picture (one out of the seven alternatives) was selected each time at random. All pictures were presented only once per session.

#### Circle Tracing Task

This task is presented in more detail in Say et al. (2011). As a brief summary, for this task participants were instructed to use a stylus to trace a white circle on a gray background. The starting position for each trial was a red arc at the top of the circle. Participants had to place the stylus inside the red area and wait for a beep indicating the start of the trial. The red arc would disappear and the participant would trace the circle clockwise. For the direct condition participants were instructed to trace the circle on the tablet screen quickly and accurately until they heard a beep indicating the end of the trial. For the indirect condition a box was placed over the tablet so the participants could not see their hand; the circle was projected onto an upright screen in front of the participant (**Figure 1B**). Participants were again asked to trace the circle quickly and accurately. Each trial lasted 45 s and there were three trials per condition.

#### Combined Simon Interference – Stop-Signal Task (cSST)

This task was a combination of an alternative version of the Simon Interference task (Simon and Rudell, 1967; Simon, 1990) and the Stop-Signal task (SST) (Logan and Cowan, 1984) programmed using MATLAB. As shown in **Figure 1C**, a white arrow appeared either side of the fixation cross pointing in the left or right direction. Participants were instructed to respond with their left or right index finger to the direction in which the arrow was pointing and ignore the location of the arrow. On congruent (C) trials the arrow pointed in the same direction as the location in which it appeared on the screen. On incongruent (IC) trials the arrow pointed in opposite direction to the side of the screen it appeared. A fixation cross remained on screen throughout the task. There were 256 trials in total (128 congruent; 128 incongruent).

A stop signal was presented on 30% of trials (42 congruent, 42 incongruent). The delay between the stimulus presentation and the stop signal was dynamically adjusted throughout the task according to the staircase method to ensure the probability of inhibiting a response on a stop trial [*p*(inhibit)] converged on 0.5 by the end of the task This occurred independently for congruent and incongruent trials. If the participant successfully inhibited their response on a stop trial the stop signal delay (SSD) on the subsequent stop trial of the same condition (C or IC) would be increased by 50ms; if the participant failed to inhibit their response on a stop trial the SSD on the subsequent trial of the same condition would be decreased by 50 ms. The SSD started at 250 ms for both conditions. Each trial had a maximum length of 1250 ms. To encourage participants to respond as fast as possible, if a participant did not make a response within the given time an error message would appear: “TOO SLOW!”. A jitter interval varied the duration of fixation cross at the beginning of each trial from 800, 1000 to 1200 ms.

The presentation of arrows and the stop signal was counterbalanced for congruent vs. incongruent and left vs. right conditions. The sequence of conditions was also accounted for by counterbalancing across conditions for n-1 trials. Three pseudorandomised and counterbalanced sequences were created ensuring that the same condition was not repeated for more than three consecutive trials. These sequences were then presented counterbalanced across participants within and between sessions to control for any order effects of sequence. The task was presented in two separate blocks, to give participants a rest break midway.

#### Trail Making Test

Commonly this test is carried out using paper and pen, however, here we modified the PEBL version of the task to produce a computerized version using a touchscreen and stylus (Piper et al., 2012; Mueller and Piper, 2014; computer software^1^). The test included two parts both of which consisted of 25 circles randomly scattered over the touchscreen surface. In part A these circles were numbered 1–25 and participants were instructed to tap inside the circles in ascending order using the stylus. A line would then be drawn between consecutive numbers. In part B the circles included numbers (1–13) and letters (A–L) (**Figure 1D**). Participants were instructed to tap inside the circles in ascending order alternating between numbers and letters (1–A–2–B, etc.). Three different versions of each part were used and counterbalanced across participants and sessions. The different versions were mirrors of the original paper and pen version, created as specified in Wagner et al. (2011).

### Behavioral Analyses

Statistical Analyses were performed using the Statistical Package for the Social Sciences (IBM SPSS Package 22). All data was analyzed using mixed effects models with subject as a random effect and task-dependent factors as fixed effects. Effect sizes were calculated for all pairwise comparisons using Hedge’s g (g) (Hedges, 1981). The normality of the dependent variables and the residuals from the model were tested using the Shapiro–Wilk test. If the residuals were not normal, the variables were transformed. All RT data, as well as speed and annulus traced in circle tracing, were transformed by log_10_ before any statistical analyses were carried out. The number correct measure in the emotion recognition task was transformed using a boxcox transformation.

For each task the Pearson’s correlation coefficient (*r*) was calculated for the mean of each dependent variable for each subject for sessions 1–2 and sessions 2–3 to determine the test–retest reliability of each measure. For the measures that did not meet normality assumptions (*p* < 0.05 for the Shapiro–Wilk test) Spearman correlations were calculated. All reported *p*-values are two-tailed unless stated otherwise.

#### Emotion Recognition Task

The dependent variables were the number of emotions correctly identified (accuracy) and RT from the presentation of the face to the time a response was made. A linear mixed model was used to determine any significant main or interaction effects of session and emotion on the two dependent variables collected. The same model was then repeated to look at session effects only for the two dependent variables averaged over all negative emotions as this is often used as a summary measure for this task. Subject was included as a random factor.

#### Circle Tracing Task

The outcome measures for this task were speed (calculated as the time to complete a full circle rotation; cm/s) and length of traced annulus (cm). Length of traced annulus per trial is the most commonly used measure. Speed of tracing has also been examined, but the measure previously used was the total number of rotations completed in 45 s (Say et al., 2011). This is an approximate measure of speed and therefore does not capture the variability between different rotations. Our current measure of speed is more accurate by calculating the speed per rotation as: length of annulus traced (in cm) over time to complete the full rotation (s) averaged over all completed rotations. A linear mixed model was used to determine the effect of the fixed factors session and condition (direct or indirect) on the two outcome measures recorded. Subject was included as a random factor.

#### Combined Simon Interference – Stop-Signal Task (SST)

The main outcome measure of the Simon Interference task is the interference effect, which is the difference in RT between C and IC trials. For the SST the main outcome measure is stop-signal reaction time (SSRT). This is an estimate of how long it takes to inhibit a response from the initiation of the stop process in response to the stop-signal based on the independent horse-race model and calculated using the ‘quantile’ method (Logan and Cowan, 1984; Verbruggen and Logan, 2009).

Previous studies have demonstrated that there are longer SSRTs for IC trials when the trial is preceded by a C trial (Verbruggen et al., 2005), therefore sequential analyses were conducted on all dependent variables. These analyses look at the RT of the current trial (C or IC) when the previous trial is C.

All incorrect trials were excluded from analysis and RTs less than 250 ms or greater than 1000 ms were removed. Any SSRT values less than 50 ms were also excluded. One subject was excluded from all analyses as a large proportion of responses were excluded, suggesting that this person performed the task in a very different way than the rest.

A linear mixed model was used to determine if there were any significant main or interaction effects for the fixed factors session and block, on the dependent variables: mean RT on GO trials, interference effect on current GO trials, interference effect on sequential GO trials (trials preceded by a C trial), SSRT, SSRT interference effect on current trials and SSRT interference effect on sequential trials. Subject was included as a random factor.

#### Trail Making Task

The time it took to complete each test (part A and B separately) was recorded for each subject for each session. A linear mixed model compared the effect of the fixed factors session, map (part A or B) and version of map on completion time. Subject was included as a random factor.

## Results

### Emotion Recognition Task

**FIGURE 2:**
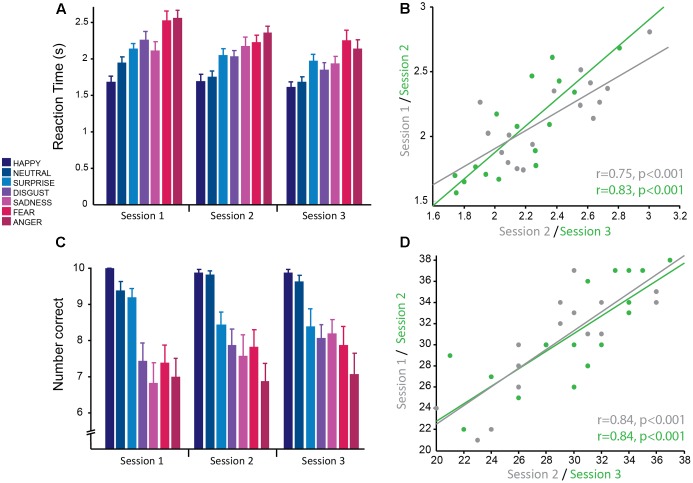
Emotion recognition task: good reliability of reaction times and accuracy across testing sessions. **(A)** Reaction time from the presentation of the stimulus to selecting the correct emotion averaged across subjects for each session. Reaction times were significantly faster for more positive emotions and slower for more negative emotions. Overall average reaction times decreased across sessions. **(B)** Significant positive correlation of mean reaction time, averaged over all negative emotions for each subject, between sessions 1 and 2 (gray) and sessions 2 and 3 (green) demonstrating good test–retest reliability. **(C)** Mean accuracy (number of faces correctly identified for each emotion) averaged across subjects for each session. Accuracy was greater for more positive emotions and less for negative emotions. Accuracy was stable across testing sessions. **(D)** Significant positive correlation of mean accuracy averaged over all negative emotions for each subject, between sessions 1 and 2 (gray) and sessions 2 and 3 (green) demonstrating good test–retest reliability.

For this task, the main dependent variables recorded were: (1) RT, the time from being presented with a face to selecting an emotion, and (2) accuracy, the number of emotional expressions of each emotion correctly recognized. Here the dependent variables were analyzed using a linear mixed effects model with the fixed effects emotion (happy, neutral, surprise, disgust, sadness, fear, and anger) and session, with subject as a random effects factor. There was a main effect of session [*F*(2,297.98) = 27.01, *p* < 0.001], whereby RT was significantly different between all pairs of sessions (all pairwise comparisons at *p* < 0.001 corrected; session 1: M ± SD = 2.15 s ± 0.47; session 2: M ± SD = 2.04 s ± 0.44; session 3: M ± SD = 1.90 s ± 0.40; **Figure 2A**). The effect size between sessions 1 and 3 was large (*g* = 0.98), whereas the effect was of medium to small size between sessions 1 and 2 (*g* = 0.47) and sessions 2 and 3 (*g* = 0.51). This suggests that there were practice effects with this task that persisted across all three sessions; however, the change between consecutive sessions was mediocre. There was no significant emotion by session interaction, which suggests that practice effects affected all emotions equally.

RT was also significantly modulated by emotion [*F*(6,297.98) = 46.40, *p* < 0.001] such that more positive emotions were more easily recognized and thus had significantly faster RTs than more negative emotions (in order from fastest to slowest; happiness: M ± SD = 1.67 s ± 0.33; neutral: M ± SD1.79 s ± 0.33; surprise: M ± SD = 2.05 ± 0.35; disgust: M ± SD = 2.04 ± 0.42; sadness: M ± SD = 2.04 ± 0.40; fear: M ± SD = 2.30 ± 0.45; anger: M ± SD: 2.35 ± 0.45; **Figure 2A**). The most positive emotions (happiness and neutral) did not have significantly different RTs (*p* > 0.05, corrected) and the most negative emotions (anger and fear) did not have significantly different RTs (*p* > 0.05, corrected). RTs for surprised, disgusted, and sad faces were also not significantly different from each other (all pairwise comparisons at *p* > 0.5, corrected), but were significantly greater than more positive emotions (happiness and neutral; all pairwise comparisons at *p* < 0.01 corrected) and less than more negative emotions (anger and fear; all pairwise comparisons at *p* < 0.01 corrected). All significant pairwise comparisons between emotions had large effect sizes (range of *g* = 0.92–2.74). This replicates previous findings with this task.

Accuracy for correctly identifying emotions was stable over sessions with no significant main effect of session (*p* = 0.34) and no significant interaction between session and emotion (*p* = 0.41). Similarly to RT, accuracy was modulated by the type of emotion [*F*(6,298.01) = 38.83, *p* < 0.001; **Figure 2C**] and was highest for positive emotions and lowest for negative emotions. Accuracy for happy (M ± SD = 9.92 ± 0.28) and neutral (M ± SD = 9.60 ± 0.74) faces was not significantly different from each other (*p* = 1, corrected), but was significantly different from all other emotions (all pairwise comparisons at *p* < 0.01, corrected; range of *g* = 0.84–2.38). The negative emotions (anger: M ± SD = 6.98 ± 2.04; fear: M ± SD = 7.69 ± 1.95; disgust: M ± SD = 7.79 ± 1.71; and sadness: M ± SD = 7.52 ± 2.13) had the lowest accuracy and were not significantly different from each other (all pairwise comparisons at *p* > 0.1, corrected), but were significantly different from the more positive emotions (all pairwise comparisons at *p* < 0.001, corrected; range of *g* = 1.52–2.37). Accuracy for surprised faces was moderate (M ± SD = 8.67 ± 1.52) and was significantly different from all other emotions (all pairwise comparisons at *p* < 0.02, corrected). There was a medium effect size for the difference in accuracy between surprise and the negative emotions fear (*g* = 0.68), disgust (*g* = 0.72) and sadness (*g* = 0.79), whereas there was a larger effect size for the emotions anger (*g* = 1.21), neutral (*g* = 0.84), and happy (*g* = 1.16). This replicates previous findings with this task. The large effect sizes between emotions suggest that these are robust effects.

The main outcome measure used to test for striatal impairment in clinical populations for this task is RT and accuracy in response to negative emotions combined (anger, fear, disgust, and sadness). RT for this combined measure showed a similar main effect of session [*F*(2,30) = 13.28,*p* < 0.001]. Pairwise *post hoc* comparisons showed that the difference in RT between sessions 1 and 2 trended toward significance (*p* = 0.081 corrected; *g* = 0.81) and there was a significant difference in RT between sessions 2 and 3 (*p* = 0.025 corrected; *g* = 0.96) and sessions 1 and 3 (*p* < 0.001 corrected; *g* = 1.77; session 1: M ± SD = 2.35 ± 0.32 s; session 2: M ± SD = 2.19 ± 0.33 s; session 3: M ± SD = 2.02 ± 0.36 s). Again, this demonstrates clear practice effects with this task that continue for multiple testing sessions. Accuracy for negative emotions combined also showed a significant main effect of session [*F*(2,30) = 4.93, p = 0.014]. Pairwise comparisons revealed no significant difference between session 1 and 2 (*p* = 0.236 corrected; *g* = 0.63) and sessions 2 and 3 (*p* = 0.60 corrected; *g* = 0.45), but a significant difference between sessions 1 and 3 (*p* = 0.012 corrected; *g* = 1.08; session 1: M ± SD = 28.63 ± 4.40; session 2: M ± SD = 30.13 ± 4.75; session 3: M ± SD = 31.19 ± 4.99). Highlighting once more that accuracy has less prominent practice effects, compared to RT.

Correlational analyses were conducted on this summary measure for negative emotions to determine how reliable this measure was across sessions. Pearson’s correlations revealed that RTs had high test–retest reliability (session 1–2: *r* = 0.75, *p* < 0.001; session 2–3: *r* = 0.83, *p* < 0.001; **Figure 2B**). As the accuracy data was not normally distributed, Spearman’s correlational analyses were used for this data. The results show accuracy also had high test–retest reliability (session 1–2: *r* = 0.84, *p* < 0.001; session 2–3: *r* = 0.84, *p* < 0.001; **Figure 2D**).

In summary, the emotion recognition task produces two outcome variables, RT and accuracy for negative emotions. Although both are highly correlated within subjects over multiple testing sessions, and thus show high test–retest reliability, there is an issue of practice effects for both variables, but more so for RT. Therefore it is imperative, if this task is to be used to track clinical impairment over time, to use a control group and include a familiarization session.

### Circle Tracing Task

**FIGURE 3:**
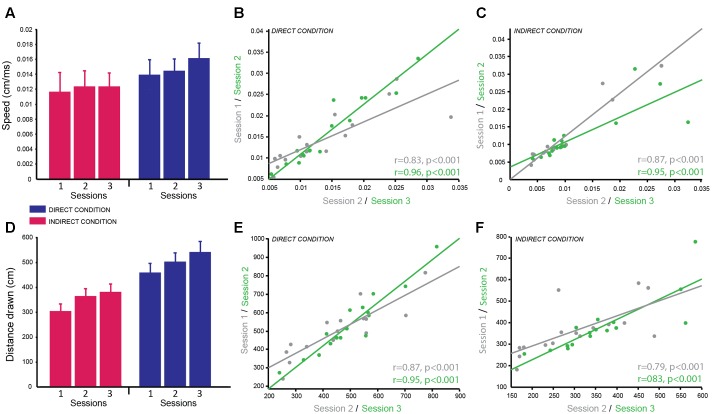
Circle Tracing Task: good reliability of speed of tracing and distance measured across testing sessions. **(A)** Mean speed of tracing (cm/s) for the indirect condition (pink) compared to the direct condition (blue) across sessions. **(B,C)** Significant positive correlation of overall mean speed of tracing for each subject between sessions 1 and 2 (gray) and sessions 2 and 3 (green) for both the direct **(B)** and indirect **(C)** conditions demonstrating good test–retest reliability. **(D)** Mean distance traced (cm) for the indirect condition (pink) and the direct condition (blue) across sessions. **(E,F)** Significant positive correlation of mean distance traced for each subject between sessions 1 and 2 (gray) and sessions 2 and 3 (green) for both the direct **(E)** and indirect **(F)** conditions demonstrating good test–retest reliability.

The dependent variables recorded for the circle tracing task were speed per trial (cm/s) and the length of the traced annulus (cm) for the direct and indirect tracing conditions. A linear mixed effects model with session and condition as fixed effects factors and subject as a random factor was used to determine the reliability of these variables across time and the difference in performance between the direct and indirect conditions. Subjects were significantly faster for the direct condition (M ± SD = 0.015 cm/s ± 0.007) compared to the indirect condition (M ± SD = 0.012 cm/s ± 0.007) as demonstrated by a significant main effect of condition for the log-transformed speed per trial [*F*(1,74) = 51.92, *p* < 0.001; *g* = 1.46; **Figure 3A**]. Speed per trial was not stable across sessions as shown by a significant main effect of session [*F*(2,74) = 10.38, *p* < 0.001]. Pairwise comparisons revealed a significant decrease in speed from session 1 to session 2 (*p* = 0.009 corrected; *g* = 0.77), which then plateaued between session 2 and session 3 (*p* = 0.50 corrected; *g* = 0.34; session 1: M ± SD = 0.010 ± 0.0062 cm/s; session 2: M ± SD = 0.012 ± 0.0071 cm/s; session 3: M ± SD = 0.013 ± 0.0073 cm/s). There was no significant interaction between condition and session (*p* = 0.48).

Distance traced was analyzed with the same linear mixed model including log-transformed speed as a covariate to account for the speed vs. accuracy trade-off. As expected there was a significant main effect of condition [*F*(1,69.34) = 16.21, *p* < 0.001; *g* = 2.28] as subjects traced a greater distance in the direct condition (M ± SD = 500.50 cm ± 148.37) compared to the indirect condition (M ± SD = 347.20 cm ± 104.04; **Figure 3D**). There was no significant main effect of session (*p* = 0.64) and no significant interaction (*p* = 0.97) suggesting the distance traced was stable over time for both conditions.

Correlational analyses were conducted separately for the two dependent variables and two experimental conditions to determine how reliable these measures were over time. Both dependent variables were well correlated across sessions for both conditions demonstrating high test–retest reliability (speed per trial direct condition: session 1–2 = *r* = 0.83, *p* < 0.001; session 2–3 = *r* = 0.96, *p* < 0.001; **Figure 3B**; speed per trial indirect condition: session 1–2 = *r* = 0.96, *p* < 0.001; session 2–3 = *r* = 0.91, *p* < 0.001; **Figure 3C**; distance traced direct condition: session 1–2 = *r* = 0.87, *p* < 0.001; session 2–3 = *r* = 0.95, *p* < 0.001; **Figure 3E**; distance traced indirect condition: session 1–2 = *r* = 0.79, *p* < 0.001; session 2–3 = *r* = 0.83, *p* < 0.001; **Figure 3F**). Speed per trial for the indirect condition was not normally distributed therefore Spearman’s correlation analysis was used here.

In summary, subjects were significantly slower to complete the task and traced a smaller distance in the indirect condition compared to the direct condition, which replicates previous findings of this same task. These variables showed high test–retest reliability between sessions as seen from the high correlation coefficients. However, similar to the emotion recognition task, the speed to complete the task was vulnerable to practice effects therefore when measuring behavioral changes in a patient population a control group will be required or a pre-baseline training session should be included. In contrast, by using speed as a covariate when analyzing the distance traced data this variable appeared to be stable across sessions and highly reliable making it a useful and robust variable for multiple testing.

### Combined Simon-Stop-Signal Task (SST)

**FIGURE 4:**
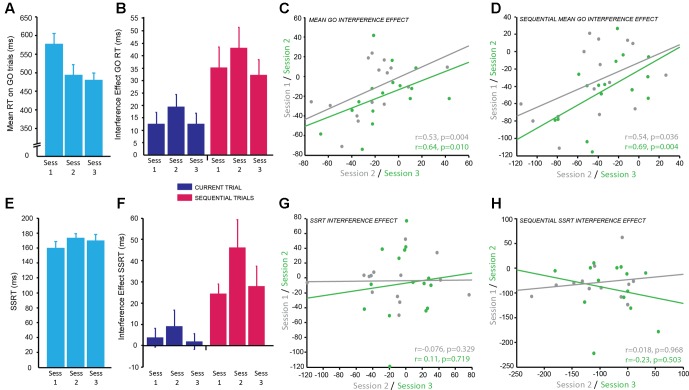
Combined Simon-Stop-Signal Task: the Simon interference effect showed moderate reliability across testing sessions but the stop-signal reaction time (SSRT) interference effect did not. **(A)** Mean reaction time (ms) on GO trials; significantly decreased from session 1 to 2 with practice effects and then plateaued between sessions 2 and 3. **(B)** The interference effect for GO trials (difference in RT between congruent and incongruent conditions) across sessions for current trials (blue) and sequential trials (pink). The interference effect was significantly greater for sequential trials (pink) compared to current trials (blue). The interference effect was stable across sessions. **(C,D)** Significant positive correlation between the mean interference effect for each subject between sessions 1 and 2 (gray) and sessions 2 and 3 (green) for current trials **(C)** and sequential trials **(D)** demonstrating moderate test–retest reliability. **(E)** Mean SSRT across sessions; no significant change in SSRT with session. **(F)** The combined Simon-Stop-Signal measure – the SSRT interference effect – for current trials (blue) and sequential trials (pink). The SSRT interference effect for current trials (blue) was not significantly different from zero, however, the SSRT interference effect for sequential trials (pink) was significant. This remained stable across sessions. **(G,H)** No relationship between the SSRT interference effect in session 1 vs. 2 (gray) and session 2 vs. 3 (green) for current trials **(G)** and sequential trials **(H)**. The SSRT interference effect showed poor test–retest reliability.

The main dependent variable recorded in this task was RT: the time to make a button press (using the Left or Right index finger) from the presentation of the stimulus. To determine the reliability of RT on GO trials (trials with no stop-signal), we examined differences across sessions and blocks. There was a significant main effect of session [*F*(2,167.25) = 42.51, *p* < 0.001]. *Post hoc* pairwise comparisons established that RT significantly decreased between sessions 1 and 2 (*p* < 0.001 corrected), but remained stable between sessions 2 and 3 (*p* = 0.911 corrected; *g* = 0.18; **Figure 4A**; session 1: M ± SD = 576.23 ± 116.34 ms; session 2: M ± SD = 492.89 ± 115.59 ms; session 3: M ± SD = 478.22 ± 80.59 ms). There was also a significant main effect of block [*F*(1,167.04) = 7.19, *p* = 0.008] showing that RT significantly increased in block 2 (*p* = 0.008 corrected; *g* = 0.39; M ± SD = 517.41 ± 98.31 ms) compared to block 1 (M ± SD = 492.90 ± 77.26 ms). Subjects appeared to slow down their RT throughout the task most likely to improve accuracy of stopping on trials in which the stop-signal was presented. However, the effect size of this comparison was small.

In this task the direction of arrows were either congruent (C) or incongruent (IC) with the side of the screen on which they were displayed. The Simon Interference effect is the difference in mean RT between C and IC conditions on GO trials. The overall effect of condition, i.e., the Simon Interference effect, was significantly different from zero (IC–C; M ± SD = 14.45 ± 20.95 ms; *t*(15) = 2.95, *p* = 0.010; *g* = 0.65), which suggests on average RT was faster on C trials compared to IC trials. Previous research has suggested this congruency effect is greater when only analyzing trials preceded by a C trial: sequential trials. Indeed, there was a significant congruency effect for sequential trials (c_IC-c_C; M ± SD = 36.98 ± 29.08 ms; *t*(15) = 4.84, *p* < 0.001; *g* = 1.21) and this was significantly greater than the interference effect based on the condition of the current trial [*t*(15) = -5.83, *p* < 0.001; *g* = 0.85; **Figure 4B**]. To identify if this congruency effect was stable we examined differences across blocks within a session and across testing sessions for both congruency measures (current and sequential trials). The mean congruency effect across GO trials on current and sequential trials showed no significant effect of session (IC–C: *p* = 0.31; c_IC-c_C: *p* = 0.28), no significant effect of block (IC–C: *p* = 0.067; c_IC-c_C: *p* = 0.40), and no significant interaction (IC–C: *p* = 0.87; c_IC-c_C: *p* = 0.35). This suggests the Simon congruency effect for RT on GO trials was very stable and not susceptible to practice effects.

The main dependent variable for the SST is the SSRT: the average time taken to inhibit a response to the stop signal. To identify the reliability of this measure within and across sessions the same mixed effects model was used. There was no significant main effect of session (*p* = 0.064), although this was trending toward significance, no significant main effect of block (*p* = 0.76), and no significant interaction (*p* = 0.79; **Figure 4E**). This suggests the SSRT was stable over time.

The aim of combining the Simon and the SSTs together was to produce a more sensitive measure of response inhibition by calculating the Simon congruency effect for the SSRT. There was no significant congruency effect for SSRT collapsed across sessions when accounting for the condition of the current trial (IC–C; M ± SD = 0.036 ± 0.13; *t*(15) = 1.11, *p* = 0.29; *g* = 0.27), however, there was a significant congruency effect for SSRT on sequential trials preceded by a C trial (c_IC-c_C; M ± SD = 0.19 ± 0.22; *t*(15) = 3.52, *p* = 0.003; *g* = 0.82). This combined SSRT congruency effect for sequential trials also appeared stable across sessions as measured using the mixed effects model; there was no significant effect of session (*p* = 0.35), no significant effect of block (*p* = 0.61), and no significant interaction (*p* = 0.38; **Figure 4F**).

In order to determine how reliable the congruency effect was over time within subjects for both GO trials and SSRT, correlational analyses between sessions were conducted. The congruency effect for GO RT on current trials showed a positive correlation with moderate reliability for sessions 1 and 2 (*r* = 0.53, *p* = 0.04) and slightly higher reliability for sessions 2 and 3 (*r* = 0.64, *p* = 0.01; **Figure 4C**). The weaker correlation between sessions 1 and 2 is likely due to practice effects. A similar result was found for sequential trials with moderate reliability for sessions 1 and 2 (*r* = 0.54, *p* = 0.036) and higher reliability for sessions 2 and 3 (*r* = 0.69, *p* = 0.004; **Figure 4D**). In contrast, despite appearing stable over time, the congruency effect for SSRT showed no correlation between sessions for both current trials (session 1–2: *r* = -0.076, *p* = 0.329; session 2–3: *r* = 0.11, *p* = 0.719; **Figure 4G**) and sequential trials (session 1–2: *r* = -0.018, *p* = 0.968; session 2–3: *r* = -0.23, *p* = 0.503; **Figure 4H**) and therefore poor test–retest reliability. It may be the case that a greater number of sessions is needed for this to become a reliable measure. SSRT alone had a moderate correlation between sessions 1 and 2 (*r* = 0.64, *p* = 0.018) and sessions 1 and 3 (*r* = 0.66, *p* = 0.014). However, there was no significant correlation between sessions 2 and 3 (*p* = 0.369). It is possible that participants used a different strategy in session 2 which affected the reliability of this measure.

In summary, the main markers of inhibitory control in the Simon Interference task (1: the difference in GO RT between current C and IC trials; 2: the difference in GO RT between C and IC trials preceded by a C trial) showed moderate test–retest reliability. Despite the absence of significant differences between the sessions, the correlation between sessions was only moderate suggesting that more sessions might be necessary for participants to stabilize their performance. The main marker of inhibitory control from the SST, the SSRT, showed similar results, whereby despite the absence of significant session effects, the correlation between sessions was moderate to low. Therefore, a greater number of sessions is needed to conclude whether SSRT is a reliable measure. Finally, the combined Simon Interference and Stop-Signal measure of inhibitory control, the congruency effect for SSRT was not well correlated across sessions, therefore showed poor test–retest reliability and is not a reliable measure for use in clinical populations.

### Trail Making Task

**FIGURE 5:**
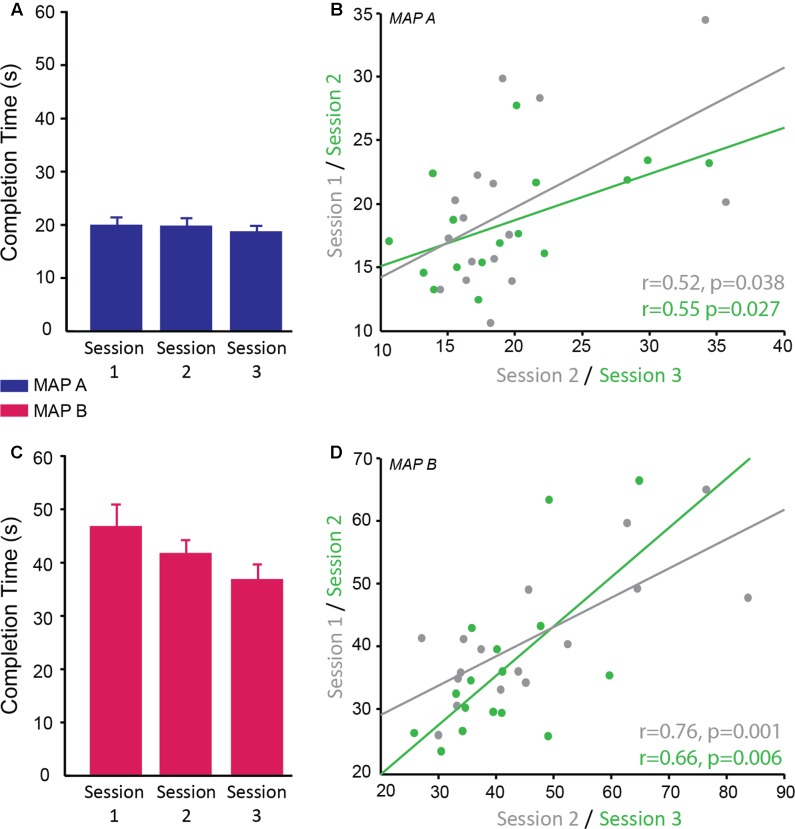
Reliability of completion time on the trail making test. **(A,C)** Completion time (s) of trail map A (**A**; blue) and map B (**C**; pink) across sessions. There was no significant interaction between map and session therefore it cannot be concluded that map A showed different reliability across sessions compared to map B; however, qualitatively the data suggests map B may be more susceptible to practice effects than map A. **(B,D)** Significant positive correlation of completion time on trail map A **(B)** and trail map B **(D)** between sessions 1 and 2 (gray) and sessions 2 and 3 (green).

The time to complete the Trail Making Task was recorded for two difficulty levels (map A and map B) of which there were three versions of each. A linear mixed model was used to determine the reliability of completion time over sessions, the difference in completion time between maps A and B and whether map version affected the behavioral result. There was a significant main effect of map [*F*(1,69) = 371.99, *p* < 0.001; *g* = 0.389] with subjects taking longer to complete map B (M ± SD = 41.52 ± 12.04 s) compared to map A (M ± SD = 19.32 ± 4.88 s) confirming that map B was more difficult as expected. There was no significant difference in performance between the different versions of each map (*p* = 0.810) and no significant interaction between map and version (*p* = 0.843), therefore different versions can be used for multiple testing sessions. There was a significant main effect of session [*F*(2,69) = 4.57, *p* = 0.014]. *Post hoc* pairwise comparisons revealed a significant difference between sessions 1 and 3 (*p* = 0.011 corrected; *g* = 0.75), with participants being faster at the third session compared to the first session. There was no significant difference between session 1 and 2 (*p* = 0.654; *g* = 0.31) and between session 2 and 3 (*p* = 0.246; *g* = 0.44; session 1: M ± SD = 33.21 ± 10.19 s; session 2: M ± SD = 30.52 ± 7.92 s; session 3: M ± SD = 27.54 ± 7.84 s; **Figures 5A,C**). There was no significant interaction between map and session (*p* = 0.172).

Correlational analyses suggested that map A was less reliable than map B. Map A showed a moderate correlation between sessions 1 and 2 (*r* = 0.52, *p* = 0.038) and sessions 2 and 3 (*r* = 0.55, *p* = 0.027; **Figure 5B**), while map B showed higher correlation between sessions 1 and 2 (*r* = 0.76, *p* = 0.001) and sessions 2 and 3 (0.66, *p* = 0.006; **Figure 5D**).

In summary, subjects found map B more difficult than map A as reflected by longer completion time for map B. Map A and map B showed moderate test–retest reliability with map B appearing to be better for multiple testing potentially due to the greater difficulty level. The use of different versions did not have a significant effect on performance, therefore are appropriate for longitudinal testing in order to minimize practice effects. Despite the fact that different versions were used and participants saw a different version at each visit, there were some moderate effects of practice. As with the other tasks in this study, the significant effect of practice means a control group is required if testing this longitudinally in a clinical population.

## Discussion

This study aimed to determine the test–retest reliability of outcome measures from a number of cognitive tasks commonly used to assess cognitive impairment in clinical populations such as those with HD and PD. The reliability of a measure is affected by the degree of measurement noise, which is assumed to vary across trials and visits, and the magnitude of the true signal, which is assumed to be reliable across repeated measurements. Different outcome measures have different signal magnitude and susceptibility to noise, therefore establishing which tasks are reliable over time is essential if these tasks are to be used to track clinical progression. Four cognitive tasks (a combined Simon Stop-Signal Task; a modified emotion recognition task; a circle tracing task; and the trail making test) were tested in a sample of healthy subjects across three testing sessions. Practice effects were seen between sessions 1 and 2 across all tasks for the majority of dependent variables, particularly reaction time variables. Good test–retest reliability was seen for RT and accuracy for negative emotions in the emotion recognition task and speed and distance traced in the circle tracing task (*r* > 0.7). Completion time of the trail making test A and B and the Simon interference effect for GO trials and SSRT from the combined-Simon-Stop-Signal task showed moderate test–retest reliability (*r* > 0.5). However, the combined SSRT interference effect showed poor test–retest reliability (r < 0.3).

The emotion recognition task and the circle tracing task appeared to produce the most reliable and replicable outcome variables. This is supported by previous studies that have shown high reliability (*r* > 0.85) for measures of the emotion recognition task (Stout et al., 2012). RT in this task was subject to practice effects, which did not plateau within the three sessions measured suggesting a greater number of sessions is required before this measure becomes stable; however, despite this, responses were very reliable between sessions. Conversely, accuracy for correctly identifying negative emotions was very stable over time, which supports previous research that has shown this to be the more reliable measure (Stout et al., 2014). These results are particularly encouraging as the emotion recognition task used here was a modified electronic version of the task. In previous versions of the task participants either name the emotion out loud, in which case the response is recorded by a voice key, or selected using a stylus, in the case of a tablet. In conditions such as PD and HD, where participants suffer from motor impairment and speech difficulties, using a voice key is not recommended, because participants often make accompanying sounds prior to speaking, which can trigger the voice key. Responding using a button box or tablet is therefore preferable. However, when using a standard tablet for the task, without an extended monitor, the presentation of the face and the emotion choices is crammed and it is difficult to arrange the buttons equidistant, so as to obtain unbiased RT measures for each trial. To overcome this problem we modified the presentation of the task to include a tablet, where the participant would make a response using a mouse button, and an external monitor where the participant would view the pictures of the faces (see section “Materials and Methods”). All buttons were arranged in equal distance around the start position of the cursor, to avoid positional bias in RTs. As expected we were able to replicate the negative emotion recognition effect for both the number of correct responses and RTs, i.e., participants made more mistakes and were slower for negative emotions than the happy or neutral emotion. We also showed that both RT and accuracy for negative emotions were reliable across sessions in this modified, more optimal set up of the task.

Additionally, the outcome measures of the circle tracing task were very reliable over time. This is particularly encouraging for the speed measure, which was calculated differently to previous studies using this task. Here the measure of speed was more accurate by calculating the actual speed per rotation compared to previous studies which have used an approximate measure of number of rotations completed in a given time (Say et al., 2011). We demonstrate that this new method is accurate and reliable over time. This measure was, however, susceptible to practice effects over the first two sessions, but these plateaued between sessions 2 and 3. Distance traced on the other hand, after adjusting for speed, remained stable across all sessions. Both variables showed strong positive correlations across sessions suggesting that these are reliable and useful variables for measuring task performance longitudinally in clinical populations.

Similar results regarding the reliability of dependent variables were also seen for the trail making test and the combined Simon-Stop-Signal task, however, these were not as reliable as the measures from the previously discussed tasks. Completion time in the trail making test reduced across sessions 1 and 3 and showed moderate correlation between sessions for both maps with map B appearing slightly more reliable. This is perhaps not surprising given the nature of the task; as this task is quite cognitively demanding and the timing measure relatively crude there can be a lot of intra-individual variability, which decreases the reliability of this measure over multiple testing sessions. Alternatively, the emotion recognition task and the circle tracing task have less intra-individual variability making these tasks more reliable.

In the combined Simon-Stop-Signal task, the Simon Interference effect (both current and sequential) was stable across sessions and showed moderate correlation between sessions. This was similar to the mean SSRT, which was also stable across sessions and showed moderate correlation between sessions. It is interesting to note that for these cases of RT, despite the absence of significant session differences, the correlation across sessions only showed moderate correlation (between 0.5 and 0.7). This suggests there was individual variability over time making such RT measures less reliable across sessions. It is important that measures have both high within-subject correlation across sessions and a lack of session effects to be deemed to have good test–retest reliability as defined in this study.

For the combined Simon-Stop-Signal task, we used a tracking method to modulate the stop-signal delay based on accuracy and controlled for task difficulty throughout, therefore it could be suggested that the second half of trials would provide a more reliable measure than the first half or the total. However, there was no significant difference in SSRT between the first and second half of the task (no main effect of block) in this study. Indeed, a previous study found that the most reliable SSRT estimates are generated from averaging over all trials and using a lenient outlier criteria, which is the approach we used here (Congdon et al., 2012). The study by Congdon et al. (2012) also suggested that data should be pooled across multiple runs of this task in order to get the most reliable estimate of SSRT. As we only had one run per testing session (divided into two blocks to include a rest break) we could not measure this in the current study. However, if multiple runs are carried out at each session, the mean between session estimates of SSRT may show higher reliability and be more optimal for tracking disease progression, therefore should be considered when using this task to measure clinical populations.

In the current study, we additionally analyzed a novel measure produced from the combined Simon-Stop-Signal task: the SSRT interference effect. A previous study, Jahfari et al. (2011), examined effective connectivity between areas of the cortex and basal ganglia during a similar version of the combined Simon-Stop-Signal task and found differential patterns of connectivity for the interference effect on successful and failed stop trials. This suggests that quantifying the combined effect of inhibiting a response to the stop signal and the more prepotent interference effect may offer another, potentially more sensitive measure, of response inhibition which can be used to track disease progression in clinical populations. However, in the current study this effect showed large inter-individual variability across sessions, despite the absence of significant session differences, and therefore showed poor test–retest reliability. Previous studies have shown higher reliability for SSRT in tasks without the tracking procedure, therefore this may be a more favorable method for this combined task. In addition, difference scores will inherently show less reliability across time points than means generated from large samples of data; therefore, as this variable requires a combination of two difference scores there may need to be a larger increase in the number of trials in order to counteract this and produce a more reliable measure.

An important issue with using cognitive tasks in longitudinal studies is the presence of practice effects, which were prominent in the current study. For most of the measure in this study there was only a significant difference in performance between sessions 1 and 2, therefore a single familiarization session may be sufficient to eliminate practice effects. It has been shown previously that using alternate forms of a task may be useful in attenuating practice effects (Beglinger et al., 2005). Indeed, for the trail-making test, when the same version of this task is administered in a longitudinal study, participants become familiar with the position of the numbers and letters and therefore it is particularly vulnerable to practice effects. A modified version of the original Trail Making Task was previously tested using paper and pencil and no version effects were found, therefore the modification was deemed suitable for longitudinal studies (Wagner et al., 2011). In this study we implemented three versions of the task in PEBL and we tested the test–retest reliability of the time to complete measure, as well as whether there were any version effects. We showed moderate reliability for map A and good reliability for Map B of completion time over sessions and no significant version effects. However, the presence of practice effects, even with multiple versions of this task persisted, which suggests that multiple pre-baseline training sessions are required if this is to be used in longitudinal studies. Our findings do not support the argument that multiple versions of a task eliminate practice effects; however, the use of multiple versions here may have reduced the magnitude of these effects, but this cannot be tested in the current study.

One limitation of the current study is that the sample consisted of young, healthy and well educated participants; disease onset in patients with striatal impairment for which these tasks are often used to track disease progression is around >50 years therefore it is important that reliability studies such as this are repeated in older populations. Despite this, using a well-matched control group may still not optimally account for differences in the rate of improvement through practice between the groups tested. Age and medical health have been shown to impact practice effects (Calamia et al., 2012); and older participants and those with cognitive impairment show reduced practice effects. A longitudinal study, tracking circle tracing in HD patients demonstrated that HD patients had reduced practice effects on this task compared to healthy controls and pre-HD patients, which was explained by a lack of improvement rather than disease decline (Tabrizi et al., 2013). The inclusion of a healthy control group may not be sufficient to account for practice effects such as these. This highlights the lack of generalizability of the current findings and emphasizes the need to repeat reliability studies in specific patient populations being tested before conducting clinical trials. Moreover, the current study is limited by the small sample size; large studies would be preferable and provide more conclusive evidence regarding the reliability of the measurements. Nevertheless, the magnitude of the effect sizes provides some certainty about the observed effects and the power to detect an effect in future studies. In addition, establishing the reliability of tasks in healthy controls can help identify the tasks and measures that should be further tested in patients. Tasks which are not vulnerable to practice in healthy samples are likely to be more appropriate for testing in patients with cognitive impairment as these patients are less likely to show effects of practice compared to healthy controls. However, if a task is vulnerable to practice in healthy subjects, then the effect of practice in specific patient populations needs to quantified or assess the use of individualized pre-baseline training as the means to eliminate practice effects prior to clinical testing.

In summary, the current study tested the reliability of a number of dependent variables produced from cognitive tasks used to track striatal function over time: the circle tracing task; the emotion recognition task; the trail making test; and a combined-Simon-Stop-Signal task. Firstly, the results demonstrate that all of the tasks, in particular the circle tracing and the emotion recognition task, produce reliable outcome measures over repeated sessions. However, not all variables produced from these tasks were reliable and stable over time; care should be taken when selecting which variables will be used to track clinical progression. In particular, SSRT interference was highly variable within-subjects across sessions. Secondly, the current study highlights the variables with higher within-individual variability in these tasks. This emphasizes the need for appropriate pre-baseline training in studies tracking performance longitudinally and further emphasizes the need for control groups when tracking progression in clinical groups to account for these practice effects.

## Author Contributions

CP: collected and analyzed data, wrote the paper. DL: analyzed data, provided important edits on the paper. ST: conception of research idea, provided important edits on the paper. MP: conception of research idea, designed study, analyzed data, provided important edits on the paper.

## Conflict of Interest Statement

The authors declare that the research was conducted in the absence of any commercial or financial relationships that could be construed as a potential conflict of interest.

## Footnotes

1 http://pebl.sourceforge.net
